# Limited Predictive or Prognostic Role of Tumor-Infiltrating Tissue-Resident Memory CD8 T Cells in Patients with Hepatocellular Carcinoma Receiving Immunotherapy

**DOI:** 10.3390/cancers13205142

**Published:** 2021-10-14

**Authors:** Ying-Chun Shen, Ching-Ping Yeh, Yung-Ming Jeng, Chiun Hsu, Chih-Hung Hsu, Zhong-Zhe Lin, Yu-Yun Shao, Li-Chun Lu, Tsung-Hao Liu, Chien-Hung Chen, Ann-Lii Cheng

**Affiliations:** 1 Department of Medical Oncology, National Taiwan University Cancer Center, Taipei 10672, Taiwan; yingchunshen@ntu.edu.tw (Y.-C.S.); hsuchiun@ntu.edu.tw (C.H.); zzlin7460@ntu.edu.tw (Z.-Z.L.); 2 Department of Oncology, National Taiwan University Hospital, Taipei 10002, Taiwan; 113297@ntuh.gov.tw (C.-P.Y.); chihhunghsu@ntu.edu.tw (C.-H.H.); yuyunshao@ntu.edu.tw (Y.-Y.S.); lichun@ntuh.gov.tw (L.-C.L.); 017027@ntuh.gov.tw (T.-H.L.); 3 Graduate Institute of Oncology, College of Medicine, National Taiwan University, Taipei 10055, Taiwan; 4 Department of Pathology, National Taiwan University Hospital, Taipei 10002, Taiwan; chengym@ntu.edu.tw; 5 Department of Internal Medicine, National Taiwan University Hospital, Taipei 10002, Taiwan; chenhcc@ntuh.gov.tw; 6 Department of Internal Medicine, National Taiwan University Hospital Yunlin Branch, Yunlin 64041, Taiwan

**Keywords:** tissue-resident memory CD8 T cells, hepatocellular carcinoma, immune checkpoint blockade, immunotherapy

## Abstract

**Simple Summary:**

Total tumor-infiltrating CD8 T cells inconsistently correlate with the efficacy of immune checkpoint blockade (ICB) in hepatocellular carcinoma. Tumor-infiltrating CD8 tissue-resident memory T cells (T_RM_) are considered a surrogate of tumor-specific T cells and correlated better with survival in patients with melanoma, non–small-cell lung cancer, head and neck cancer or bladder cancer who received ICB. However, in this study, compared with total tumor-infiltrating CD8 T cells, tumor-infiltrating CD8 T_RM_ cells failed to provide additional advantages in predicting the efficacy of ICB-based immunotherapy in patients with hepatocellular carcinoma.

**Abstract:**

Purpose: Tumor-infiltrating tissue-resident memory CD8 T cells (CD8 T_RM_; CD103+ CD8+) are considered tumor-specific and may correlate better with the tumor response to immune checkpoint blockade (ICB). This study evaluated the association of tumor-infiltrating CD8 T_RM_ and their subsets with the efficacy of immunotherapy in patients with advanced hepatocellular carcinoma (HCC). Experimental Design: Consecutive HCC patients who received ICB in prospective trials were analyzed. Formalin-fixed paraffin-embedded tumor sections were stained for DAPI, CD8, CD103, CD39, programmed cell death-1 (PD-1), and programmed cell death ligand 1 (PD-L1) using a multiplex immunohistochemical method. The densities of CD8 T cells, CD8 T_RM_, and CD39+ or PD-L1+ subsets of CD8 T_RM_ were correlated with tumor response and overall survival (OS). Results: A total of 73 patients were identified, and 48 patients with adequate pretreatment tumor specimens and complete follow-up were analyzed. A median of 32.7% (range: 0–92.6%) of tumor-infiltrating CD8 T cells were T_RM_. In subset analyses, 66.6% ± 34.2%, 69.8% ± 33.4%, and 0% of CD8 T_RM_ cells coexpressed CD39, PD-L1, and PD-1, respectively. The objective response rates for CD8 T cell-high, CD8 T_RM_-high, CD39+ CD8 T_RM_-high, and PD-L1+ CD8 T_RM_-high groups were 41.7%, 37.5%, 37.5%, and 29.2%, respectively. Patients with CD8 T cell-high, but not those with CD8 T_RM_-high, CD39+ CD8 T_RM_-high, or PD-L1+ CD8 T_RM_-high, tumors, had significantly prolonged OS (*p* = 0.0429). Conclusions: Compared with total tumor-infiltrating CD8 T cells, tumor-infiltrating CD8 T_RM_ or their subsets failed to provide additional advantages in predicting the efficacy of immunotherapy for HCC.

## 1. Introduction

CD8 T cell-infiltrated tumors are generally considered to be more immunogenic and more likely to respond to immune checkpoint blockade (ICB) [1]. However, total tumor-infiltrating CD8 T cells did not correlate well with the objective tumor response to nivolumab in patients with advanced hepatocellular carcinoma (HCC; CheckMate 040 study) [2]. Recent studies have demonstrated that bystander CD8 T cells targeting tumor-irrelevant antigens are abundant among tumor-infiltrating CD8 T cells in multiple cancer types [3,4]. This may partly explain why total tumor-infiltrating CD8 T cells did not correlate well with the response to ICB in patients with HCC and highlights the need to analyze tumor-specific T cells selectively.

Tumor-infiltrating tissue-resident memory CD8 T cells (T_RM_; expressing the tissue residency marker CD103) are considered to be highly tumor-specific and are correlated better with survival in patients with various types of cancers [5,6,7,8,9]. This subpopulation of tumor-infiltrating CD8 T cells is retained in the tumor microenvironment following initial activation and expansion and plays an essential role in tumor-immune equilibrium [10] and tumor surveillance [11,12]. Tumor-infiltrating CD8 T_RM_ are characterized by the higher clonality of T-cell receptor repertoires [7,13] and can efficiently kill autologous tumor cells in a major histocompatibility complex class I-dependent manner [6,14]. By contrast, compared with their non-T_RM_ counterparts, tumor-infiltrating CD8 T_RM_ cells more frequently express inhibitory molecules such as CD39 (the rate-limiting enzyme in the conversion of ATP to immunosuppressive adenosine) [15], programmed cell death-1 (PD-1), cytotoxic T lymphocyte antigen-4, lymphocyte activation gene-3, and T-cell immunoglobulin and mucin domain-3 while maintaining effector functions [7,16]. Tumor-infiltrating CD8 T_RM_ cells were increased in patients with melanoma or non-small cell lung cancer who responded to ICB, but not in those nonresponders [5,17]. These findings suggest that tumor-infiltrating CD8 T_RM_ cells are responsive to ICB-invoked immune regulation. Moreover, the CD39 coexpression of tumor-infiltrating CD8 T_RM_ cells has been linked to higher tumor specificity and reactivity [3,14]. Therefore, tumor-infiltrating CD8 T_RM_ cells or their subsets, instead of total tumor-infiltrating CD8 T cells, may exhibit a better correlation with the efficacy of ICB-based immunotherapy in patients with HCC.

The current study characterized CD8 T_RM_ cells in the tumor microenvironment of HCC and investigated the association between CD8 T_RM_ cells and their subsets expressing CD39 or PD-1/PD-L1 signaling and the efficacy of ICB-based immunotherapy in patients with advanced HCC.

## 2. Materials and Methods

### 2.1. Patients

Patients with advanced HCC who met the following criteria were included in this study: (1) received ICB-based immunotherapy in prospective clinical trials from August 2015 to March 2019; (2) had high-quality pre-immunotherapy archived tumor tissues with viable tumor parts, as assessed by a senior independent pathologist; and (3) had complete clinical follow-up information and an evaluable tumor response to ICB-based immunotherapy according to Response Evaluation Criteria in Solid Tumors (RECIST; version 1.1) [18]. Clinical information including patients’ characteristics and their tumors, immunotherapy regimens, prior systemic therapy, date of immunotherapy initiation, the best response according to RECIST (version 1.1), and date of death was obtained from electronic medical records. Objective responses included complete response (CR) and partial response (PR). Overall survival (OS) was defined as the time from the initiation of immunotherapy to death due to any cause or the last follow-up. This study was approved by the Research Ethics Committee of National Taiwan University Hospital (202001070RIND) and conducted in compliance with the Declaration of Helsinki and other ethical guidelines.

### 2.2. Multiplex Fluorescent Immunohistochemical Staining

Hematoxylin/eosin (H/E)-stained slides of formalin-fixed paraffin-embedded (FFPE) tumor blocks were evaluated by an independent pathologist (YMJ). The block with largest area of viable tumors was selected for sectioning at a thickness of 5 μm. The viable tumor parts were marked on H/E slides. Selected FFPE sections were deparaffinized, rehydrated, antigen retrieved, and stained using a customized multiplex fluorescent immunohistochemical (IHC) panel (Opal 7-color manual IHC kit; Akoya, Marlborough, MA, USA) according to the manufacturer’s instructions. The primary antibodies used were CD8 (clone: C8/144B; 1:400) from DAKO (Santa Clara, CA, USA), CD39 (clone: polyclonal; 1:100) from Sigma (St. Louise, MO, USA), CD103 (clone: EPR4166 [2]; 1:100) from Abcam (Cambridge, UK), and PD-1 (clone: EH33; 1:100) and PD-L1 (clone: E1L3N; 1:200) both from Cell Signaling (Danvers, MA, USA). Spectral 4′,6-diamidino-2-phenylindole was used for nuclear counterstaining. FFPE sections from a tonsillectomy specimen and a PD-L1-high non–small-cell lung cancer specimen were used for the optimization of the staining protocol and as positive controls for PD-1 and PD-L1 staining. FFPE sections from a known CD8 T_RM_-rich HCC tumors were included in each staining batch to detect any batch effects as a quality control measure.

### 2.3. Multispectral Fluorescent Imaging and Analysis

Visualization of multiplex fluorescent imaging was performed using Vectra Polaris Automated Quantitative Pathology Imaging Systems (Perkin Elmer, Hopkinton, MA, USA). Color separation, tissue and cell segmentation, and cell phenotyping were performed using inForm Software v2.4.2 (Perkin Elmer, Hopkinton, MA, USA). Multispectral regions of interest (200× magnification field) were randomly selected from the viable tumor part of each slide—as many as possible. The densities (number/mm^2^) of CD8 T cells (CD8+), CD8 T_RM_ (CD103+ and CD8+), CD39+ CD8 T_RM_ (CD39+, CD103+, and CD8+), PD-1+ CD8 T_RM_ cells (PD-1+, CD103+, and CD8+), and PD-L1+ CD8 T_RM_ cells (PD-L1+, CD103+, and CD8+) in each area of interest were automatically quantitated under the supervision of a skilled researcher (CPY) and a senior pathologist (YMJ), who were blinded to the response status. The average density of each cell type for each tumor was calculated. The median value of immune cell density of interest among all tumors was used to divide tumors into “high (infiltration)” and “low (infiltration)” groups.

### 2.4. Statistical Analyses

The Mann–Whitney test was performed to compare binary outcome variables. Fisher’s exact test was used when proportions were compared between binary variables. Nonparametric Spearman correlation was performed to measure the degree of association between two variables. The log-rank (Mantel–Cox) test was used to compare OS. Above analyses were conducted in GraphPad Prism (GraphPad Software, La Jolla, CA, USA). Cox regression analyses were performed to evaluate the risk factors for death and were conducted in IBM SPSS Statistics version 28.0.0.0 (New York, NY, USA).

## 3. Results

### 3.1. Baseline Characteristics and Treatment Outcomes of Enrolled Patients

A total of 73 patients with advanced HCC who received ICB-based immunotherapy in global open-label clinical trials were identified. Of these 73 patients, 25 were subsequently excluded due to the following reasons: no archived tumor specimens in 18; scant tumor cells in the FFPE slide in 5; no tumor part in the FFPE slide in 1; and death before tumor assessment in 1 (Figure S1). Finally, 48 patients were included in this study, and their baseline characteristics are shown in Table 1. Most of them were men (42, 87.5%) and HBV carriers (36, 75%). All of them had a Child–Pugh Classification A liver function and Eastern Cooperative Oncology Group (ECOG) performance status of 0–1 according to the eligibility criteria of clinical trials (Table S1). A total of 41 (85.4%) and 20 (41.7%) patients had extrahepatic metastasis and vascular invasion, respectively. Half of them had never received first-line sorafenib for advanced HCC. Most (31, 64.6%) of them received ICB-based combination therapy.

Fifteen (31.3%) of these patients were responders (4 showed CR and 11 showed PR). The only difference in baseline features between responders and nonresponders (patients with stable disease or progressive disease) was vascular invasion (20% vs. 51.5%; *p =* 0.0401; Table 1). During a median follow-up of 30.7 months, the median OS for all patients, responders, and nonresponders was 35, not reached, and 16 months, respectively (Figure S2).

### 3.2. Characteristics of Archived Tumors and Their Multispectral Image Acquisition

The characteristics of archived tumors and their multispectral image acquisition are shown in Table S2. Most (40, 83.3%) of the archived tumors were primary hepatic tumors, and 21 (52.5%) of them were obtained from previous hepatectomy. The median ages of surgical and biopsy specimens were 17.2 and 0.9 months, respectively (*p* = 0.002). On average, 28.4 and 7.3 multispectral regions of interest were selected from each surgical and biopsy specimen, respectively. The representative figures are shown in Figure S3.

### 3.3. Tumor-Infiltrating CD8 T_RM_ Cells

**Figure 1:**
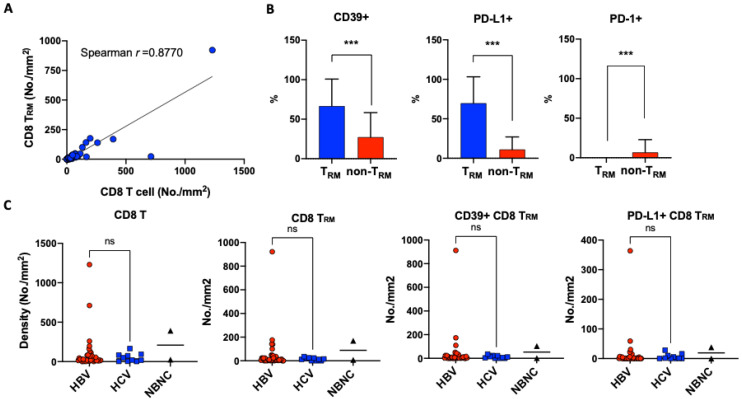
CD8 T_RM_ cells in the tumor microenvironment of HCC. (**A**) Correlation between the densities of tumor-infiltrating CD8 T cells and densities of tumor-infiltrating CD8 T_RM_ cells; (**B**) Coexpression of CD39, PD-L1, and PD-1ontumor-infiltrating CD8 T_RM_ cells and CD8 non-T_RM_ cells; ***: *p* < 0.001; (**C**) Correlation between densities of tumor-infiltrating CD8 T cells, CD8 T_RM_ cells, CD39+ CD8 T_RM_ cells, or PD-L1+ CD8 T_RM_ cells and the etiologies of HCC. Statistical analyses were performed only for comparisons between HBV and HCV; ns: not statistically significant.

**Figure 2:**
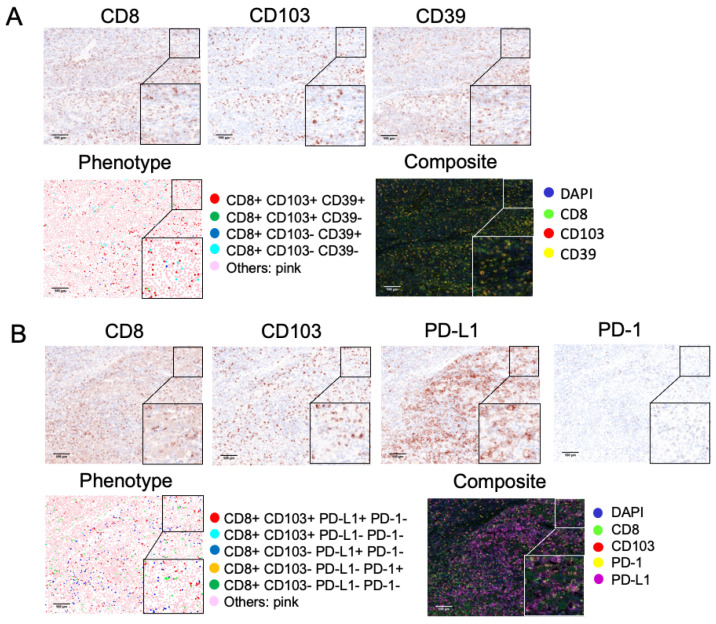
Representative images of tumor-infiltrating CD8 T_RM_, CD39+ CD8 T_RM_, and PD-L1+ CD8 T_RM_ cells. (**A**) CD8, CD103, and CD39 staining images and the composite image; (**B**) CD8, CD103, PD-L1 and PD-1 staining images and the composite image. PD-1 was primarily expressed on non-CD8 T cells. Each is shown at 200× magnification field (400× magnification field for the right lower corner square).

The density of tumor-infiltrating CD8 T_RM_ cells correlated well with that of tumor-infiltrating CD8 T cells (Spearman *r* = 0.8770; *p* < 0.0001; Figure 1A). A median of 32.7% (range: 0%–92.6%) of tumor-infiltrating CD8 T cells were T_RM_. Compared with non-T_RM_ CD8 T cell counterparts, CD8 T_RM_ cells more frequently coexpressed CD39 or PD-L1, but not PD-1 (Figure 1B). On average, 66.6% ± 34.2%, 69.8% ± 33.4% and 0% of tumor-infiltrating CD8 T_RM_ cells coexpressed CD39, PD-L1, and PD-1, respectively. The representative figures are shown in Figure 2. The density of tumor-infiltrating CD8 T_RM_ cells or their CD39+ or PD-L1+ subsets did not significantly correlate with the etiologies of HCC (Figure 1C).

### 3.4. Correlations with Efficacy of Immunotherapy

**Figure 3:**
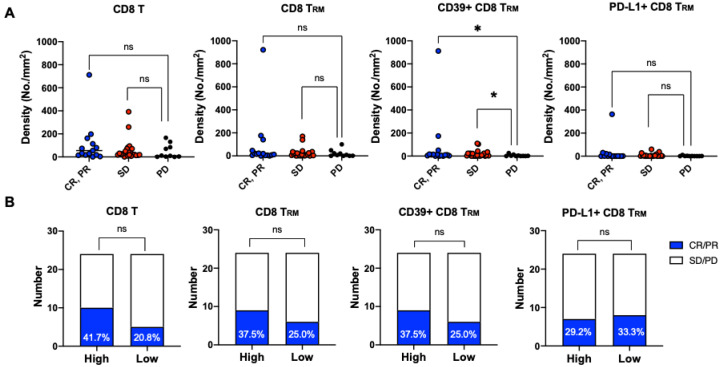
Correlations of CD8 T_RM_ cells or their subsets with response to ICB-based immunotherapy. (**A**) Correlations among densities of tumor-infiltrating CD8 T cells, CD8 T_RM_ cells, CD39+ CD8 T_RM_ cells, or PD-L1+ CD8 T_RM_ cells and the best response according to RECIST version 1.1; (**B**) Numbers of responders (CR/PR) and nonresponders (SD/PD) according to the infiltration levels of CD8 T cells, CD8 T_RM_ cells, CD39+ T_RM_ cells, and PD-L1+ T_RM_ cells (high vs. low). CR, complete response; PR, partial response, SD, stable disease; PD, progressive disease; ns, not statistically significant; *, *p* < 0.05 but > 0.01.

**Figure 4:**
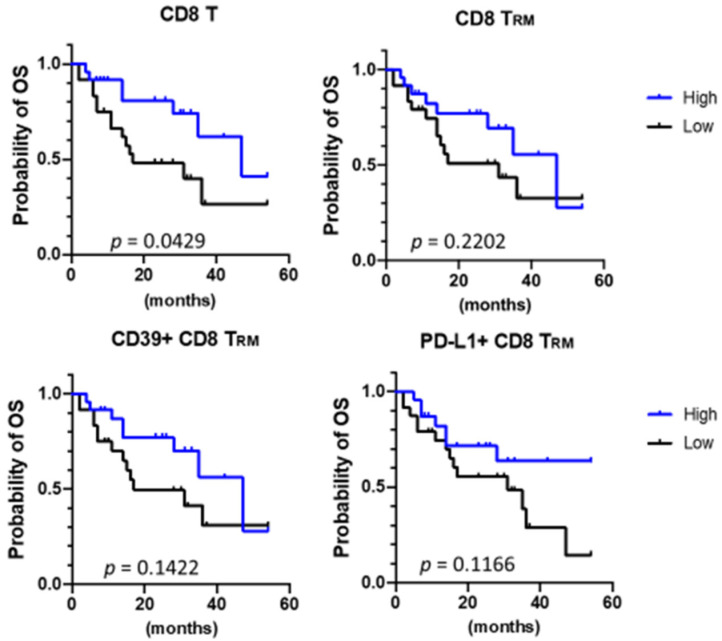
Correlations of CD8 T_RM_ cells or their subsets with overall survival. Overall survival (OS) curves according to the infiltration levels of CD8 T cells, CD8 T_RM_ cells, CD39+ CD8 T_RM_ cells, and PD-L1+ CD8 T_RM_ cells are shown (Kaplan-Meier method). Significance was tested using log-rank (Mantel-Cox) test.

The densities of tumor-infiltrating CD8 T, CD8 T_RM_, CD39+ CD8 T_RM_, and PD-L1+ CD8 T_RM_ cells are shown by the best response in Figure 3A. Objective responses were associated with higher densities of tumor-infiltrating CD39+ CD8 T_RM_ cells (*p* = 0.04 for both CR/PR vs. stable disease and CR/PR vs. progressive disease comparisons). The objective response rates of patients with CD8 T cell-high, CD8 T_RM_-high, CD39+ CD8 T_RM_-high, and PD-L1+ CD8 T_RM_-high tumors were 41.7%, 37.5%, 37.5%, and 29.2%, respectively (Figure 3B). Patients with CD8 T cell-high tumors were associated with significantly prolonged OS (*p* = 0.0429). Patients with CD8 T_RM_-high, CD39+ CD8 T_RM_-high, and PD-L1+ CD8 T_RM_-high tumors showed a trend of better survival; however, the finding was not statistically significant (Figure 4). The baseline characteristics were not different between patients with CD8 T cell-high tumors and those with CD8 T cell-low tumors, likewise between patients with CD8 T_RM_-high tumors and those with CD8 T_RM_ low tumors.

### 3.5. Prognostic Factors in HCC Patients Receiving Immunotherapy

Univariate Cox regression analysis revealed that vascular invasion posed a higher risk for death (hazard ratio: 2.934; *p* = 0.020) while objective response and high CD8 T cell density posed lower risks for death (hazard ratio: 0.084, and 0.398, respectively; *p* = 0.002 and 0.048, respectively). However, high CD8 T cell density was no longer an independent prognostic factor after controlling all variables in multivariate Cox regression analysis (Table 2).

## 4. Discussion

The role of tumor-infiltrating CD8 T_RM_ cells, a potential surrogate of tumor-specific CD8 T cells, in predicting the efficacy of immunotherapy in cancer patients remains elusive. The current study correlated the densities of tumor-infiltrating CD8 T_RM_ cells, their subsets, and total CD8 T cells with the efficacy of ICB-based immunotherapy in clinical trial patients with advanced HCC. Our data revealed that neither tumor-infiltrating CD8 T_RM_ nor its CD39+ or PD-L1+ subset provided additional advantages over total tumor-infiltrating CD8 T cells in predicting the efficacy of ICB-based immunotherapy in patients with advanced HCC. However, neither total tumor-infiltrating CD8 T cells nor tumor-infiltrating CD8 T_RM_ cells are independent prognostic factors in HCC patients receiving ICB-based immunotherapy.

In contrast to the current study, three previous studies have demonstrated that tumor-infiltrating CD8 T_RM_ cells correlated with prolonged survival in cancer patients treated with ICB. Two of them were conducted using bulk RNA-sequencing data obtained from clinical trials or published studies of melanoma, non–small-cell lung cancer, and bladder cancer [19,20]. Another study used a multiplex fluorescent IHC method in patients with non–small-cell lung cancer [17]. Banchereau et al. [19] quantified tumor-infiltrating CD8 T_RM_ cells by using *ITGAE* (encoding CD103) gene expression, whereas Zhang et al. [20] used a CD8 T_RM_ signature consisting of *CXCR6*, *ZNF683*, and *ITGAE* genes. The *ZNF683* gene encodes a T_RM_-specific transcriptional factor; however, *CXCR6-* and *ITGAE-*encoded proteins are expressed in a wide range of immune cells such as regulator T cells, natural killer cells, and dendritic cells. Thus, the abundance of CD8 T_RM_ cells determined by either *ITGAE* gene expression or T_RM_ signature may not be specific enough for CD8 T_RM_. Corgnanc et al. [17] and the current study used the multiplex fluorescent IHC method, which is superior to the gene expression approach for its simultaneous colocalization and visualization [21]. However, our results did not show any significant correlation of tumor-infiltrating CD8 T_RM_ cells with OS in patients with HCC patients who were treated with ICB-based immunotherapy. These findings indicated that the roles of tumor-infiltrating CD8 T_RM_ cells in antitumor immunity may vary by cancer type.

CD8 T_RM_ is a heterogenous population [22]. CD39+ CD8 T_RM_ cells have been well characterized as a highly tumor-reactive subset of CD8 T_RM_ cells in non–small-cell lung cancer [6], head and neck cancer [14], and endometrial cancer [23], but they have never been investigated in HCC. A recent study reported that the frequency of CD39+ CD8 T cells well correlated with tumor mutation burden as well as high-affinity neoantigen burden in HCC [24]. Moreover, sorted tumor-infiltrating CD39+ CD8 T cells from human HCC, but not CD39^−^ CD8 T cells, elicited high-affinity neoantigen-specific T-cell responses upon ex vivo neoantigen peptide stimulation. This finding strongly suggests that neoantigen-specific CD8 T cells are enriched in CD39+ CD8 T cells. According to our data, 66.2% ± 33.1% of tumor-infiltrating CD39+ CD8 T cells were T_RM_**.** Therefore, tumor-infiltrating CD39+ CD8 T_RM_ cells in HCC are considered highly tumor-specific and responsive. The densities of CD39+ CD8 T_RM_ cells were significantly higher in responders (Figure 3A); however, high infiltration of CD39+ CD8 T_RM_ cells failed to predict an objective response or prolonged overall survival in our patients. We hypothesized that the effector functions of CD39+ CD8 T_RM_ cells in patients with HCC may be limited by local cytokine milieu [25] or metabolic fitness [26]. Therefore, future studies should focus on the functional characterization of CD39+ CD8 T_RM_ cells to better understand their role in anti-tumor immunity in HCC.

Aside from the heterogeneity of CD8 T_RM_ cells, two other reasons may also explain the limited predictive or prognostic value of tumor-infiltrating CD8 T_RM_ cells in HCC patients receiving immunotherapy. First and most importantly, tumor-infiltrating CD8 T_RM_ cells are not a good surrogate of tumor-specific CD8 T cells in HCC. The theory of differential tumor specificity between tumor-infiltrating CD8 T_RM_ cells and CD8 non- T_RM_ cells was initially established in lung [6] and breast [7] cancers, which were not virus-associated cancers. However, approximately 80% of HCC arise from virus-infected liver. Virus-specific CD8 T cells may be coincidently present in the tumor microenvironment of HCC. This argument is supported by a recent study, in which the investigators identified not only tumor-specific CD8 T_RM_ cells but also HBV-specific CD8 T_RM_ cells from HCC-infiltrating T cells using peptide-major histocompatibility complex tetramers and single cell RNA sequencing [27]. It indicates that tumor-infiltrating CD8 T_RM_ cells are not highly tumor-specific in HCC, especially in virus-associated HCC. Second, a significant overlap between CD8 T cell-high tumors and CD8 T_RM_-high tumors was noted in our HBV-related HCC-predominant cohort. Twenty (83.3%) out of 24 CD8 T cell-high tumors were also characterized as CD8 T_RM_-high tumors. Therefore, CD8 T_RM_ cells are less likely to provide additional advantages than total CD8 T cells in predicting the outcome of immunotherapy in our patient cohort. It is necessary to recruit more patients with non-HBV-related HCC for further validation.

Our study has several limitations. First, the sample size was relatively small, and the treatment regimens were heterogenous; however, this insufficiency may be partly alleviated by the stringency of conducting global prospective trials. Ideally, such a study should be conducted under a single large-scale clinical trial; however, pretreatment archived tumor samples are usually not absolutely required for recruitment in large-scale clinical trials. Therefore, obtaining an adequate number of archived tumors from a single clinical trial would be considerably difficult. Second, the lack of functional characterization of CD8 T_RM_ may limit the implication of the results. Third, approximately 46% of archived tumors analyzed in this study were obtained through core biopsies that were often small pieces of tissues. Thus, whether intratumor heterogeneity of CD8 T_RM_ cells and their subsets may affect the reliability of estimating the immune composition of the whole tumor by measuring such a small piece of tissue remains unclear. Nevertheless, we recently indicated that the intratumor heterogeneity of the immune tumor microenvironment may not be a major concern in HCC [28].

## 5. Conclusions

We demonstrated that tumor-infiltrating CD8 T_RM_ cells or their subsets may not have significant predictive or prognostic value in patients with advanced HCC who received ICB-based immunotherapy. Further studies are required to elucidate the contradictive roles of tumor-infiltrating CD8 T_RM_ cells in various cancer types. 

## Figures and Tables

**Table 1:** Characteristics of enrolled patients and their archived tumors.

Variable	All (*N* = 48)	CR/PR (*N* = 15)	SD/PD (*N* = 33)	*p*-Value *
**Age (years-old)**				NS
Median	63	63.9	61.9	
Range	25.2–76.9	50.2–75.9	25.2–76.9	
**Gender**				NS
Male	42	12	30	
Female	6	3	3	
**Viral status**				NS
HBV	36	12	24	
HCV	10	3	7	
Non-HBV and non-HCV	2	0	2	
**Vascular invasion**				0.0401
No	28	12	16	
Yes	20	3	17	
**Extrahepatic spread**				NS
No	7	3	4	
Yes	41	12	29	
Lung	24	6	18	
Lymph node	21	6	15	
Bone	8	3	5	
Peritoneum/pleura	5	1	4	
Adrenal gland	2	1	1	
**AFP level**				
≤400 ng/mL	30	10	20	NS
>400 ng/mL	18	5	13	
**Prior sorafenib**				NS
No	24	7	17	
Yes	24	8	16	
**Regimen of immunotherapy**				NS
Anti-PD-1 or anti-PD-L1 monotherapy	16	4	12	
Nivolumab	12	3	9	
Tislelizumab	1	0	1	
Atezolizumab	1	0	1	
Durvalumab	2	1	1	
Anti-CTLA-4 monotherapy	1	1	0	
Tremelimumab	1	1	0	
Anti-PD-1 plus anti-CTLA-4	14	5	9	
Nivolumab plus ipilimumab	8	4	4	
Durvalumab plus tremelimumab	6	1	5	
Anti-PD-L1 + Anti-glypican-3V	2	1	1	
Atezolizumab plus codrituzumab	2	1	1	
Anti-PD-L1 + Anti-VEGF	15	4	11	
Atezolizumab plus bevacizumab	15	4	11	
**Archived tumor**				NS
Surgical specimen	26	9	17	
Biopsy specimen	22	6	16	
**Time from tumor sampling to immunotherapy (month)**				NS
Median	7.2	17.2	6.4	
Range	0.2–144.4	0.4–144.4	0.2–74.8	
**Organ of specimen**				NS
Liver	40	13	27	
Lung	3	1	3	
Lymph node	2	1	1	
Bone	2	0	2	

* comparison between CR/PR and SD/PD; CR, complete response; PR, partial response; SD, stable disease; PD, progressive disease; NS, not statistically significant; HBV, hepatitis B virus; HCV, hepatitis C virus; AFP, alpha-fetoprotein; VEGF, vascular endothelial growth factor.

**Table 2:** Cox regression analysis of risk factors for death.

Variable	Univariate Analysis				Multivariate Analysis			
	HR	95% IC (Lower)	95% IC (Upper)	*p*	HR	95% IC (Lower)	95% IC (Upper)	*p*
≥63 years-old	0.559	0.231	1.353	0.197				
Male	1.206	0.348	4.176	0.767				
HBV-related	0.935	0.341	2.565	0.897				
Vascular invasion	2.943	1.184	7.312	0.020 *	6.766	1.631	28.067	0.008 *
Extrahepatic spread	1.394	0.320	6.084	0.658				
AFP >400 ng/mL	1.047	0.433	2.531	0.919				
Prior sorafenib use	0.914	0.368	2.266	0.846				
ICI-based combination	0.454	0.187	1.104	0.082				
Objective response (CR/PR)	0.084	0.018	0.393	0.002 *	0.059	0.007	0.500	0.009 *
High CD8 T cell density	0.398	0.159	0.994	0.048 *	0.974	0.193	4.922	0.975
High CD8 TRM cell density	0.574	0.238	1.387	0.218				
High CD39+ CD8 TRM cell density	0.521	0.215	1.259	0.147				
High PD-L1+ CD8 TRM cell density	0.492	0.198	1.220	0.126				

HR: hazard ratio; IC: interval of confidence; *p*: *p*-value; HBV: hepatitis B virus; AFP: alfa-fetal protein; ICI: immune checkpoint inhibitor; CR: complete response; PR: partial response; TRM: tissue-resident memory T cells; *, statistically significant (*p* < 0.05).

## Data Availability

The data presented in this study are available in this article (and supplementary material).

## Supplementary Materials

The following are available online at https://www.mdpi.com/article/10.3390/cancers13205142/s1, Figure S1: Patient selection flowchart. Figure S2: Overall survival of all patients, responders (CR/PR) and nonresponders (SD/PD). Figure S3: Selection of multispectral regions of interest. Table S1: Clinical trials of immune checkpoint blockade-based immunotherapy for advanced hepatocellular carcinoma during 2015–2019. Table S2: Characteristics of archived tumors and their multispectral imaging acquisition.

## Abbreviations

HCC	hepatocellular carcinoma
ICB	immune checkpoint blockade
T_RM_	tissue-resident memory T cells
FFPE	formalin-fixed parafilm-embedded
IHC	immunohistochemical
OS	overall survival
MHC	major histocompatibility complex
RECIST	Response Evaluation Criteria in Solid Tumors
CR	complete response
PR	partial response
H/E	hematoxylin/eosin
ECOG	Eastern Cooperative Oncology Group
