# Supplementary material for: Limited Predictive or Prognostic Role of Tumor-Infiltrating Tissue-Resident Memory CD8 T Cells in Patients with Hepatocellular Carcinoma Receiving Immunotherapy
Source: Cancers (Basel). 2021 Oct 14;13(20):5142. doi: 10.3390/cancers13205142 (PMC8533865; doi:10.3390/cancers13205142)
Supplement: Supplementary file 1 [file cancers-13-05142-s001.zip › cancers-1417104-supplementary.pdf]

cancers

MDPI

Supplementary Materials

Limited predictive or prognostic role of tumor infiltrating tissue-resident memory CD8 T cells in patients with hepatocellular carcinoma receiving immunotherapy

Ying-Chun Shen, Ching-Ping Yeh, Yung-Ming Jeng, Chiun Hsu, Chih-Hung Hsu, Zhong-Zhe Lin, Yu-Yun Shao, Li-Chun Lu, Tsung-Hao Liu, Chien-Hung Chen and Ann-Lii Cheng

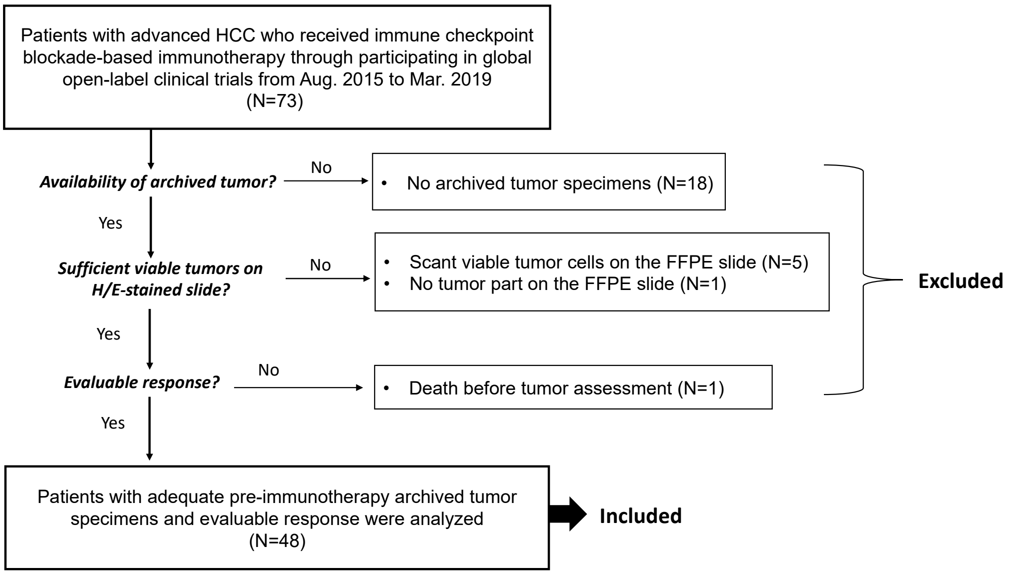

Figure S1: Patient selection flowchart.

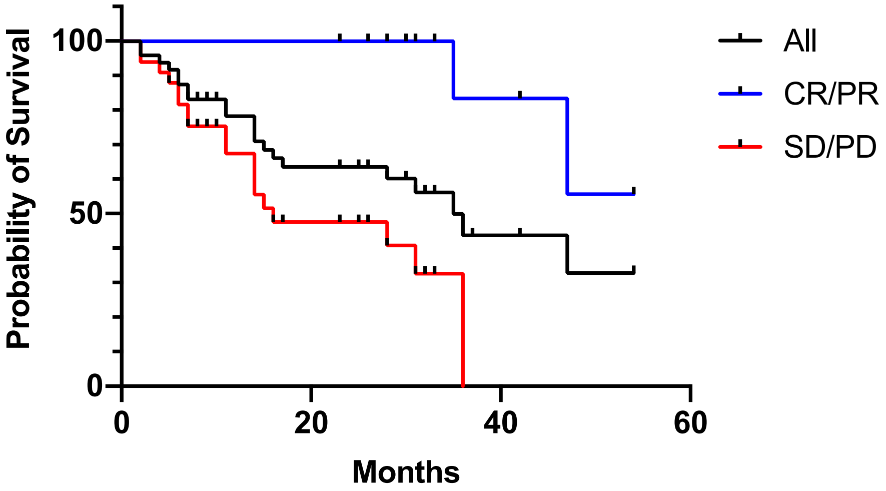

	Median OS (months)	HR (95% CI)	P-value
**CR/PR**	undefined	0.1309 (0.0543 to 0.3155)	0.0003
**SD/PD**	16		

Figure S2: Overall survival of all patients, responders (CR/PR) and nonresponders (SD/PD). CR, complete response; PR, partial response; SD, stable disease; PD, progressive disease; HR, hazard ratio; *P*-value by Log-rank (Mantel-Cox) test.

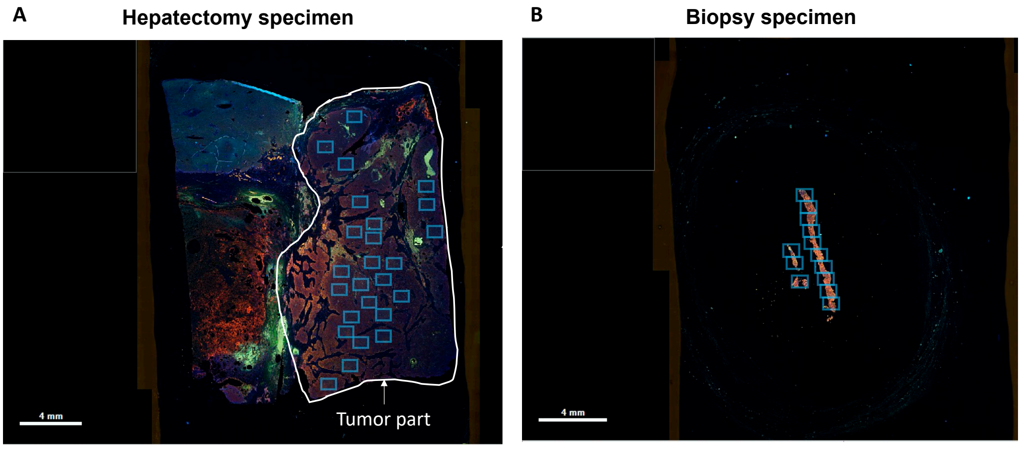

Figure S3: Selection of multispectral regions of interest. Each blue rectangle indicates a region of interest (200´ magnification field).

Table S1: Clinical trials of immune checkpoint blockade-based immunotherapy for advanced hepatocellular carcinoma during 2015-2019.

**Study name**	**Phase**	**Treatment group**	**Registration number**
CheckMate 040	I/II	-- Nivolumab<br>-- Ipilimumab<br>-- Nivolumab plus ipilimumab (3 arms)	NCT01658878
D4190C00022	II	-- Durvalumab<br>-- Tremelimumab<br>-- Durvalumab plus tremelimumab (2 regimens)<br>-- Durvalumab plus bevacizumab	NCT02519348
GO30140	Ib	-- Atezolizumab<br>-- Atezolizumab plus bevacizumab	NCT02715531
HIMALAYA	III	-- Durvalumab<br>-- Durvalumab plus t tremelimumab (2 regimens)<br>-- Sorafenib	NCT03298451
BGB-A317-208	II	-- Tislelizumab	NCT03419897
MSD/GC207JG	I	-- Atezolizumab plus codrituzumab	JapicCTI-163325

Table S2: Characteristics of archived tumors and their multispectral imaging acquisition.

	**All tumors (N=48)**	**Surgical specimens (N=26)**	**Biopsy specimens (N=22)**
**Organ**			
Liver	40	21	19
Lung	4	2	2
Lymph node	2	1	1
Bone	1	1	0
Chest wall	1	1	0

**Sample age (month)**			
Median	7.2	17.2	0.9
Range	0.2-144.4	0.4-144	0.2-60.4
**Number of multispectral regions of interest**			
Average	18.7	28.4	7.3
SD	17.2	16.5	9.1

SD: standard deviation.
